# Heavy Traffic Ahead: Car Culture Accelerates

**DOI:** 10.1289/ehp.113-a238

**Published:** 2005-04

**Authors:** Richard Dahl

As the world’s largest nation, China is drawing a lot of attention for its increased motorization rates. Although China’s rate of automobile ownership is low by the standards of the developed world, it is increasing at a very fast rate. Between the late 1970s and 2001, China’s overall fleet of motor vehicles other than two-wheelers increased 10-fold, according to an article in the 3 December 2003 issue of *Energy for Sustainable Development*.

Along the way, the Chinese government decided it wanted to develop its own auto industry. In 2001 China identified auto manufacturing as one of seven “pillar industries” of the Chinese economy and announced a five-year plan to implement a primarily domestic industry that could offer a Chinese family car at a price that would encourage widespread ownership. Between 2000 and 2004, production of passenger cars in China jumped from 605,000 to 2.33 million. On 5 February 2005, the China Federation of Machinery Industry, an industry association, forecast 20% growth for 2005 to a level that would move China past Germany into third place globally for motor vehicle production. Most industry analysts believe that the industry will continue to expand in the 15% range annually for years to come. The government has also encouraged private investments in highways to fuel a highway-building program that was already well under way. In October 2004, China’s Ministry of Communication announced that the nation’s freeways had reached 30,000 kilometers, placing it behind only the United States. Up till now, a large portion of these roads had been toll roads.

China may be getting the lion’s share of the attention, but people who follow international transportation issues say the trend in motorization is global. According to Daniel Sperling, a professor of engineering and environmental science and policy at the University of California, Davis, and director of its Institute of Transportation Studies, motorization is soaring everywhere, with the fastest growth occurring in Asia and Latin America. He says that the number of motor vehicles other than two-wheelers in the world is expected to double in the next 15 years to 1.3 billion.

“The reason motorization is spreading so rapidly is that people value mobility,” Sperling says. “But it’s also happening because a lot of regions are growing economically. We use the rule of thumb that motorization takes off when per capita incomes reach about $5,000. And many parts of the world—many cities in China, for instance—have reached that level.”

Michael P. Walsh, a transportation and environmental consultant to governments in China and other developing countries, and author of the *Energy for Sustainable Development* article, believes there’s another explanation. “Everybody, it seems to me, wants to follow the American model,” he says. “We’re held up as the country to emulate. It’s an image that people have. If you’re a modern country, you need to have lots of privately owned motor vehicles.”

Nobody doubts that there are benefits to increased mobility. But as Walsh points out, increasing numbers of automobiles and other motor vehicles in developing countries will result in deteriorating air quality, greater congestion, and poorer quality of life. It also means greater energy consumption—and for an enormously populated nation like China, which is moving rapidly and steadily toward a position of international power, the implications of growing reliance on foreign oil sources can’t be ignored.

## Impacts of Increased Motorization

Halfway during a recent talk on the current state of urban transportation in developing countries, Massachusetts Institute of Technology urban planning professor Ralph Gakenheimer turned to a series of slides to illustrate a point. “I’m about to tell you how to ride a bicycle in Shanghai when you’re making a left turn,” he told his audience. “This is only for the courageous listener.”

The slides showed extremely heavy traffic on a two-way Shanghai street divided into four lanes. Motorized vehicles occupied the inner lanes, bumper to bumper, while a dense throng of bicyclists filled the outer lanes. The Chinese system of segregating vehicles into lanes shared by similar machines is sensible to a point, Gakenheimer told the audience, but what happens if you’re a bicyclist in the outer lane and you want to take a left at the next intersection?

His series of slides provided the answer. When the traffic halts at a red light, left-turning bicyclists insinuate themselves in front of the automobiles traveling in the same direction so that when the light turns green they can dart diagonally across the intersection to their assigned lane, cutting off the automobiles coming from the opposite direction—until the automobiles force their way into the intersection to halt the bicyclists.

Similar mêlées occur throughout China, Gakenheimer says, because the numbers of cars and other motor vehicles on the nation’s roadways have been skyrocketing in recent years with no slowdown in sight. And as cars have come into repeated conflict with bicycles, increasing numbers of cities in China are taking a step that many environmentalists find troubling. “All over China, municipal governments have begun to suppress or prohibit the use of bicycles in certain places,” Gakenheimer says. “As you might imagine, this is a big controversy. Many [environmentalists] are outraged that anyone would trammel on something so affordable and environmentally sustainable as bicycles.”

The impact of increased motorization on Chinese urban air quality is still difficult to determine, Walsh says. Chinese cities have tended to have poor air quality for years, so the appearance of more motor vehicles isn’t exactly creating a new pollution problem. Instead, he says, Chinese cities are undergoing a shift from industrial pollution to motor vehicle pollution. At the same time that motor vehicles have begun to clog city thoroughfares, industry has been moving out to urban peripheries. This shift means that sulfur dioxide levels have been going down in Chinese city air, but they are being replaced by vehicle emissions including carbon monoxide and ozone-forming nitrogen oxides.

While China has greatly tightened requirements for new vehicles, the older cars on the nation’s roadways create serious pollution problems. According to the Energy Foundation, a partnership of Chinese and U.S. foundations interested in sustainable energy, recent testing shows that emission levels of Chinese autos are similar to those of cars used in the United States in the late 1960s and early 1970s; these cars emit 10–20 times more pollution than cars currently used in Western countries. According to the foundation, 40% of autos and 70% of taxis in Beijing fail to meet the most basic Western emission standards.

Increased motorization in the developing world is having social and cultural impacts on poorer societies as well. At the same time that people from rural areas continue to flood into already densely populated cities to find jobs, people whose incomes have risen to the point where they can buy cars are fleeing to the cities’ outskirts in a manner not unlike the suburbanization of American cities following World War II. The upper middle class in China already sees the car as a way to provide them more mobility, says Lee Schipper, director of research at the World Resources Institute’s Center for Transport and the Environment (EMBARQ) in Washington, D.C. “But the resulting congestion when [just] twenty percent of the daily journeys are in cars—the case in Mexico City or São Paulo today—means that nobody has more mobility.”

“It’s not that cars and two-wheelers are bad,” Schipper adds. “The problem is that they’re being put on the crowded streets so fast, everywhere, and authorities aren’t doing anything about it. There are too many of them, too soon. You can’t keep up.” In addition to rising air pollution, the glut of traffic means accident rates are high, with pedestrians and cyclists the most common victims. [For more information on the growing problem of traffic-related fatalities, see “Vehicular Manslaughter: The Global Epidemic of Traffic Deaths,” *EHP* 112:A628–A631 (2004).]

Gakenheimer notes a further pernicious effect on the individual. Public transit is the form of transportation most impacted by congestion; autos can take circuitous routes or select more accessible destinations, whereas city buses are confined to predetermined routes. And the greater the congestion, the more tempting it is to get a car. So there is what he calls a “tragedy of the commons” effect.

The effects of motorization on public transit was one of the topics covered in an article by Sperling and Eileen Claussen, president of the Pew Center on Global Climate Change, published in the spring 2004 issue of *Access* magazine. They pointed out that increased motorization also includes an explosion in two-wheel motorized vehicles in many countries, pulling riders and revenue away from public-transit systems (and, Schipper notes, clogging the streets in Asia the way cars and mini-buses do in Latin America). As a result, wrote Claussen and Sperling, “In nearly all cities worldwide, public transit is losing market share.”

The outcome in poor, densely populated cities with limited roadways for motor vehicles has been “far worse traffic congestion and pollution than exist in the United States,” wrote Sperling and Claussen, despite the fact that these cities have a fraction of the car ownership of the United States. They pointed out that the challenge of building roadways is more than just a question of economics and financing. “Only a small minority of people in the developing world own cars and benefit from massive road-building budgets,” they wrote. “In contrast, the vast majority suffer from increasing traffic congestion, noise, and pollution.” Perhaps worse, says Gakenheimer, is that this majority suffers from the separation of destinations available only to auto users—a general fragmentation of society and destination opportunities.

## The Governmental Influence

In China, a bastion of orthodox socialism not that long ago, the transition is especially jarring. In the old, purely socialist days, says Gakenheimer, the emphasis was on clustering workers, jobs, and services closely together. People lived close to their work, with commercial and business services nearby. But with the liberalization of the land market since the 1980s, that all changed.

For example, Gakenheimer says, people realized that making bicycles in the commercial center of the city had become a poor use of land, so they moved those facilities to urban peripheries where land was cheaper and they’d have more room. Then they sold the former space to buyers who used it for commercial and office use more appropriate to a modern central business district. “What this does is enormously increase the journey to work of the bicycle makers and also tends to create a central business district where there really wasn’t one before,” he says. “And the new central business district creates enormous radial commuting trip requirements.”

Another factor accelerating the pace of urban decentralization in China, Gakenheimer says, is the fact that private land ownership doesn’t exist there. Land in China is owned either by the state or by collectives, and the government has constitutionally backed power to “requisition” any land whenever it sees fit. As a result, Gakenheimer says, the suburbia that characterizes the United States is sprouting quickly in China. And as the new China begins to resemble the United States and Europe in many ways, its leaders perceive the private motor vehicle as an integral commodity. Moreover, he says, Chinese cities collect the revenue from urbanization only at the beginning of long leases. As a result, they must continue to induce urbanization to have a continual revenue stream.

But to characterize the Chinese government as being blind to the dangers of rapid motorization is inaccurate. In 2000, China banned leaded gasoline and adopted the Euro I emission standards that were enacted in Europe in the early 1990s for all new cars and trucks. Last year, the Chinese adopted the Euro II standards enacted in Europe in the mid-1990s. Some cities are going further. Walsh says that Beijing, for example, will soon require the Euro III standards, which were adopted in 2001 in Europe.

Meanwhile, China has created its first fuel economy standards for new cars, which go into effect in July 2005. In the United States, cars are required to achieve at least 27.2 miles per gallon (mpg) while SUVs and light trucks are counted under a different system (and different agency) and meet a far more lenient standard. In China, the new standards place an absolute floor on fuel economy for cars according to their weight class (there are 15 classes in China). The average weight of cars sold in China in 2003 was more than 3,000 pounds, large by developing country standards. The Chinese standards cannot prevent overall mileage from deteriorating if weight increases, Schipper says, but they may discourage manufacturers from making heavier cars that cannot meet standards for their weight classes.

Schipper says that while the speed with which China has implemented fuel standards is laudable, even more noteworthy is the fact that China is aspiring to Western standards even though its economy, on a per capita basis, is still far from Western levels. “But the question is whether these measures will be swamped by the overall increase in motorization,” he says. “And I don’t have the answer to that.”

Sperling says the escalation of motor vehicles in developing countries will only continue unless the governments in those countries intervene to halt—or at least slow down—the march to a U.S.-style transportation system featuring many personal vehicles. “The market forces are such that unless government intervenes, countries will follow that path because the elites buy the cars, the elites run the government and industry, so there’s a lot of pressure to build more roads,” he says. “It creates a spiral of more and more motorization, and it takes tremendous political leadership not to follow that path.”

## The Role of the Automakers

In 2004, a group of international automotive and energy companies known as the World Business Council for Sustainable Development issued a report called *Mobility 2030: Meeting the Challenges to Sustainability*, which detailed recommendations for achieving “sustainable mobility” on a global scale. The report defines sustainable mobility as the ability to meet the needs of society to move freely, gain access, communicate, trade, and establish relationships without sacrificing other essential human or ecological values today or in the future.

Martin Wachs, director of the Institute of Transportation Studies at the University of California, Berkeley, served as a reviewer on the project and came away from it struck by the sense of responsibility that the companies seem to hold toward auto growth in developing countries: “They acknowledged that worldwide adoption of the automobile is an environmental threat, and they are considering collectively what industries might do to create a sustainable transition to a more ‘automobilized’ world,” he says.

Wachs doesn’t believe that the report succeeds in making a convincing case for global automotive sustainability. What’s significant, though, is that it took place at all, he says. “What it represents,” he says, “is an awakening of industries, which just a few years ago might have said, ‘This isn’t our responsibility; our responsibility is to our shareholders.’ But now they’re saying that they do have responsibilities and they should be thinking in terms of fuel cells and other more energy-efficient forms of powering automobiles. So I think that’s reason to have a little bit of optimism.”

*Mobility 2030* outlines seven goals that the council believes must be met in order to achieve automotive sustainability: (1) reduce conventional vehicle emissions “so that they do not constitute a significant public health concern anywhere in the world”; (2) limit greenhouse gas emissions to sustainable levels by moving toward hydrogen and bio-based fuels; (3) significantly reduce the number of traffic-related deaths and injuries worldwide; (4) reduce traffic noise; (5) reduce traffic congestion; (6) narrow “mobility divides” between rich and poor people within countries, as well as between rich and poor countries, by improving access to transportation for poor people in rural areas; and (7) improve mobility opportunities for the general population so that people don’t need to rely on privately owned vehicles.

Sperling agrees that automakers seeking to take advantage of expanding markets in the developing world have a responsibility to ease the impacts of their products on poor societies. He also believes that private investment can be a strong tool in developing innovative transportation strategies in developing countries. Private investment, he says, and not government, accounts for most of the resources that flow from industrial to developing countries. He suggested that the Overseas Private Investment Corporation, a development agency created by the U.S. government in 1971, might create a public–private investment fund specifically targeting transportation needs in developing countries.

Schipper recently attended the 2004 Challenge Bibendum in Shanghai. This yearly meeting is attended by all the major car manufacturers and sponsored by Michelin. Schipper says he noticed the same kind of attention to the problem of global motorization that the World Business Council of Sustainable Development recognized in its *Mobility 2030* report. “They were more than willing to play ball on the clean air side,” he says. “But no individual company sees an upper limit on the number of cars that can circulate. The reality, though, is that there isn’t going to be a car in every garage because there isn’t going to be room for the garage—or the car.”

## Sensible Responses

Not all of the news about the invasion of cars and other motorized vehicles into the developing world is dire. In fact, there are several examples of strong governmental leadership taking action to soften the blow of motorization.

The island nation of Singapore has made auto ownership prohibitively expensive through the imposition of various fees, including one that must be paid just to enter an auction for a limited number of auto stickers. People who win the stickers at auction must then pay for the vehicle itself. Singapore also has an extensive public transportation system that provides access to almost everywhere on the island.

Bogotá, Colombia, has built an extremely successful bus rapid transit (BRT) system, Transmilenio, which employs large, modern buses on a dedicated thoroughfare that cuts directly through the middle of the city. According to Schipper, 5–10% of Transmilenio’s passengers are former auto users—a huge number in any new public transit system, he says.

The success of Bogotá’s BRT system and an earlier one, the pioneering BRT system in Curitiba, Brazil, in the 1970s, has drawn significant attention around the world, including the developed world. Cities that have adopted such systems include Kunming, Delhi, Los Angeles, Dublin, Paris, and most large cities in Latin America. Shanghai is currently building a BRT system and an extensive subway system with help in part from EMBARQ. And Shanghai has long imposed disincentives and restrictions on auto use, such as stiff registration fees.

Still another tactic being contemplated to reduce auto congestion in developing countries actually originated in the developed world, in the city of London, England, which pioneered the concept of “congestion pricing.” In 2003, London initiated a congestion-pricing program in which drivers had to pay a fee of £5 (about US$8) to cross a clearly marked boundary in the center city. The effect has been reduced congestion and increased public transit ridership. The fee will rise soon, says Schipper, and the area where it is payable will expand.

Luis Gutierrez, who oversees EMBARQ’s Latin American programs, says he has been talking about a congestion pricing program with officials in São Paolo, one of three Latin American cities in which EMBARQ is working to create public–private partnerships for sustainable transportation. “These kinds of policies are very simple for people to understand,” he says. “You have limited public space and a lot of demand.”

He says the idea enjoys growing public support, and city officials like it, too, because it could provide an additional source of revenue. There’s just one problem, he says: the people who own the cars—and who want to drive anywhere for as little cost as possible—are middle class, and the middle is politically powerful.

Gutierrez also says the idea of BRT systems modeled after Bogotá has become extremely popular. In 10 years, he says, Latin America will have 99 cities with populations of at least 750,000 and a total of 8,000 kilometers of heavily traveled corridors suitable for BRT systems.

Gutierrez says the problem of rapidly increasing motorization is the same everywhere, and that governments in Latin America are responding to privately owned cars in much the same way that Asian governments are. They see them as contributors to economic development—or at least as a sign of it.

But Gutierrez senses that pressure is building from a public that is growing tired of increased congestion, rising air pollution, and too many traffic accidents. Furthermore, he believes another change may be occurring. “I think that the middle class, who think having a car is equivalent to freedom, are having to modify their thoughts,” he says. “They’re finding the reality is that having a private car doesn’t mean they have the freedom to move from one place to another very fast.”

But increasing traffic congestion itself is a testament to the strength of demand for ownership of one’s own car, just like in the United States. Sperling says that developing countries and cities often lack the money, expertise, and political will to tackle the problem. “There are a lot of tools available—BRT, car sharing, even congestion pricing,” he says. “But they’re all difficult to implement. You have pricing, land use management, different ways of organizing public transport. But none of them are easy to do.”

In Sperling and Claussen’s *Access* article, they presented the case for stronger U.S. action to help developing countries achieve sustainable transportation strategies through various kinds of loans, foundation support for sending U.S. experts to assist governments in developing countries, educational programs, and the involvement of the automotive and energy industries, who have significant stakes in the long-term outcome of global motorization. They argued that the U.S. withdrawal from the Kyoto Protocol has undermined American credibility that the nation is serious about the issue of greenhouse gases. And while the United States can do a great deal to foster sound sustainable transportation programs in other countries, they suggested that it could exert far more global influence by pushing for laws to reduce greenhouse gas emissions in U.S. cars, because, they wrote, the United States “to a large degree drives the pace and direction of technology development worldwide.”

They concluded their article by sounding an alarm: “(T)here can be no doubt that the developing world is racing to repeat the developed world’s transportation history. There can be no doubt that the undesirable effects associated with that history will mitigate the many associated benefits.”

As motor vehicles proliferate in the developing world, the challenge of achieving an acceptable trade-off between economic benefits and environmental impacts is formidable. “There’s reason to be hopeful,” says Wachs. “But as the automobile becomes much more standard in places like India and China, the threat to a sustainable environment is substantial.”

## Figures and Tables

**Figure:**
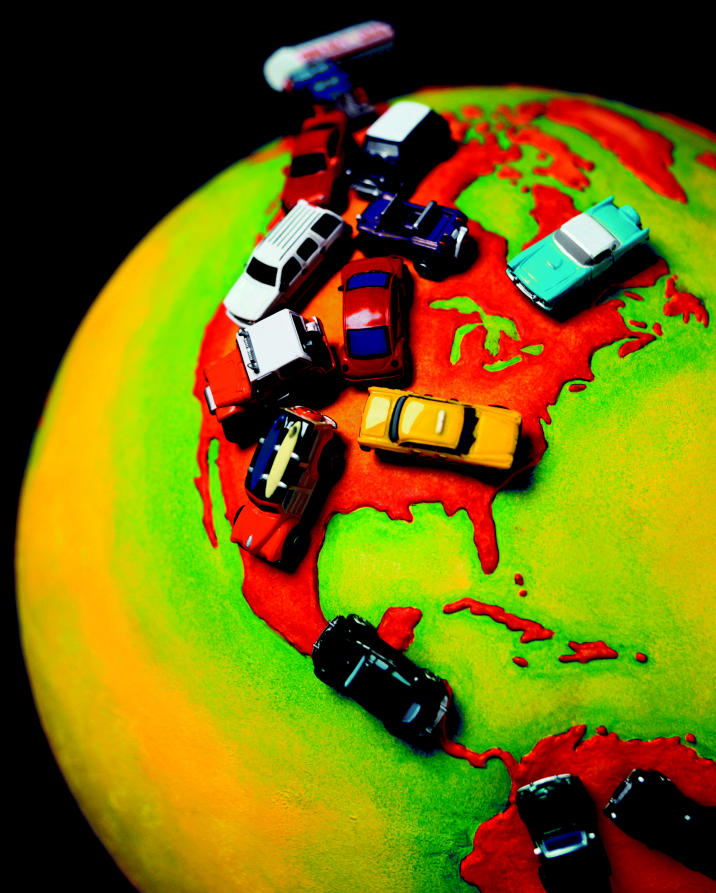


**Figure:**
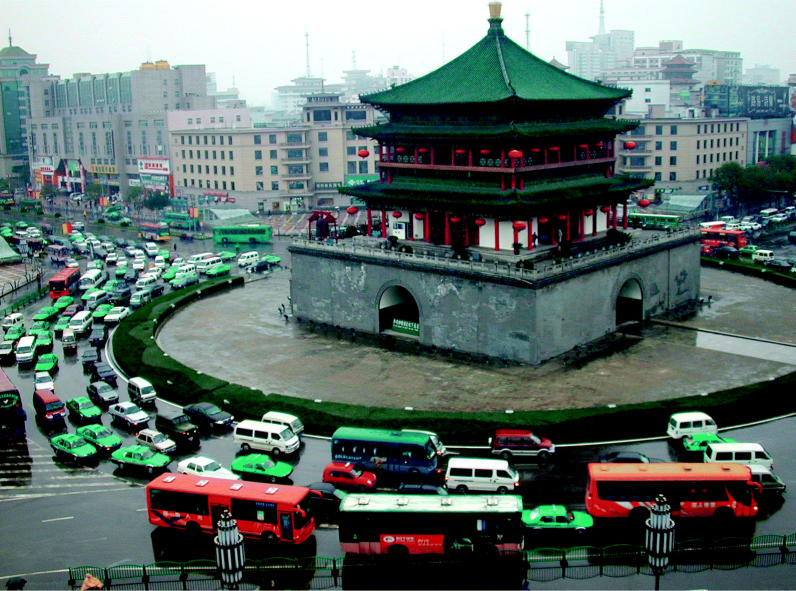
Vicious circle? Traffic around the famous bell tower in Xian, China, is typical of many urban Chinese cities, where automobiles are providing both increasing mobility and worsening air pollution.

**Figure:**
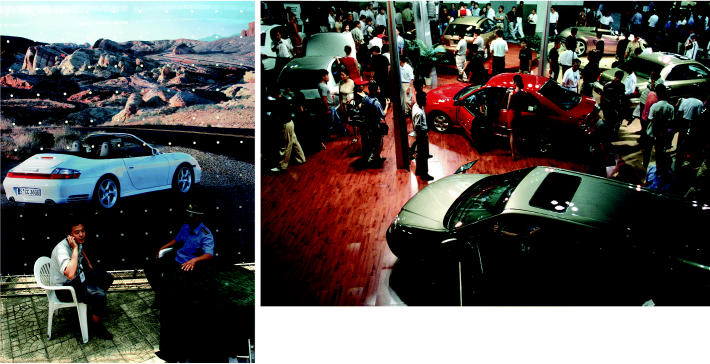
American export? The American obsession with cars seems to be taking hold overseas, as evidenced by an advertisement in Beijing (left) and the crowd eyeing the newest models during a car show in Shanghai (above).

**Figure:**
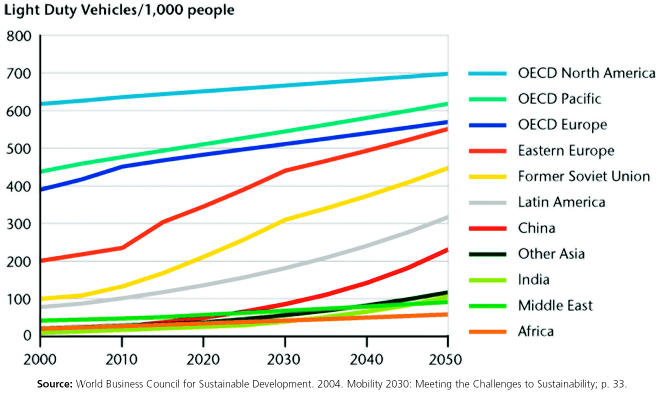
Projected growth in light duty vehicle ownership

**Figure:**
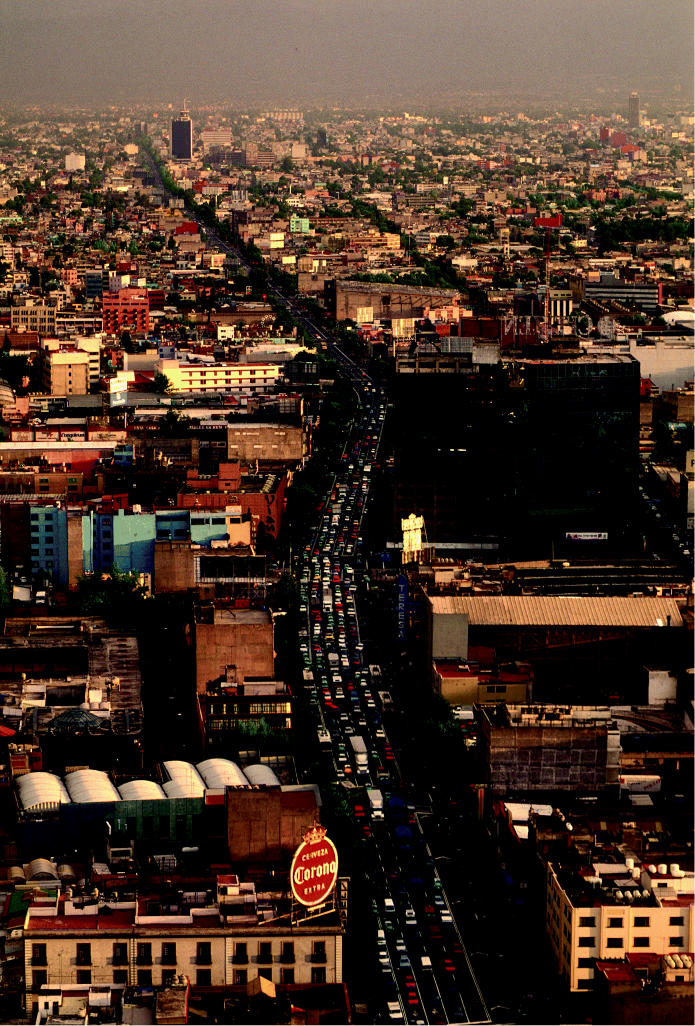
Where cities grow, cars follow Downtown traffic snakes through Mexico City. Latin America is second only to China in projected growth of personal transport activity, largely by car.

**Figure:**
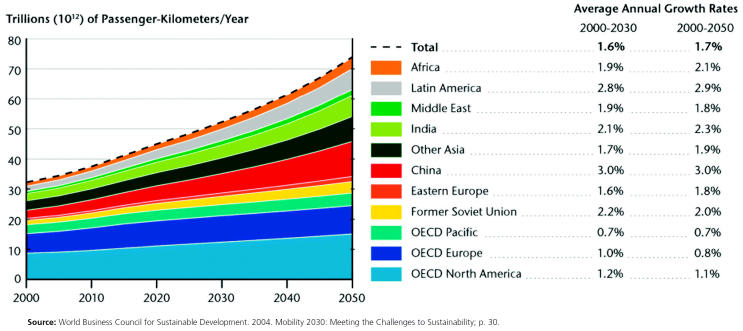
Personal transport activity by region

**Figure:**
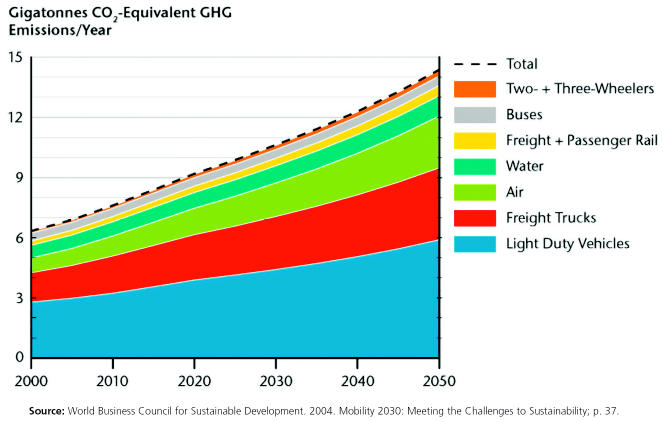
Transport-related well-to-wheels CO_2_ emissions by mode, 2000–2005

**Figure:**
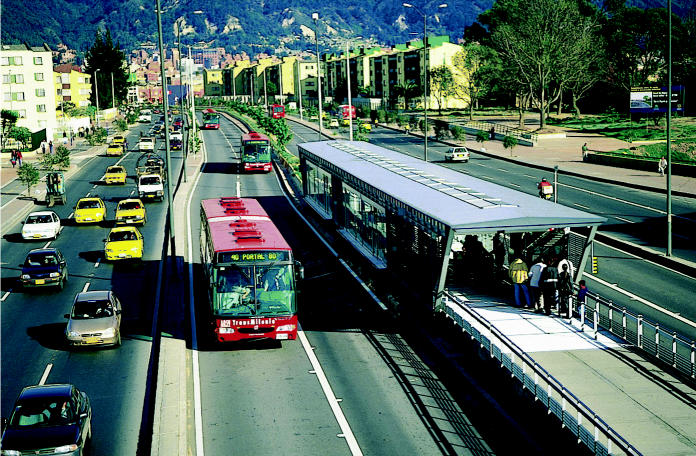
Fast progress The Transmilieno bus rapid transit system in Bogotá, Colombia, runs in separate lanes down the center of the city’s main arteries. The buses, which can carry 780,000 people a day, considerably outpace cars and save people an average of 300 hours of commuting time annually.

**Figure:**
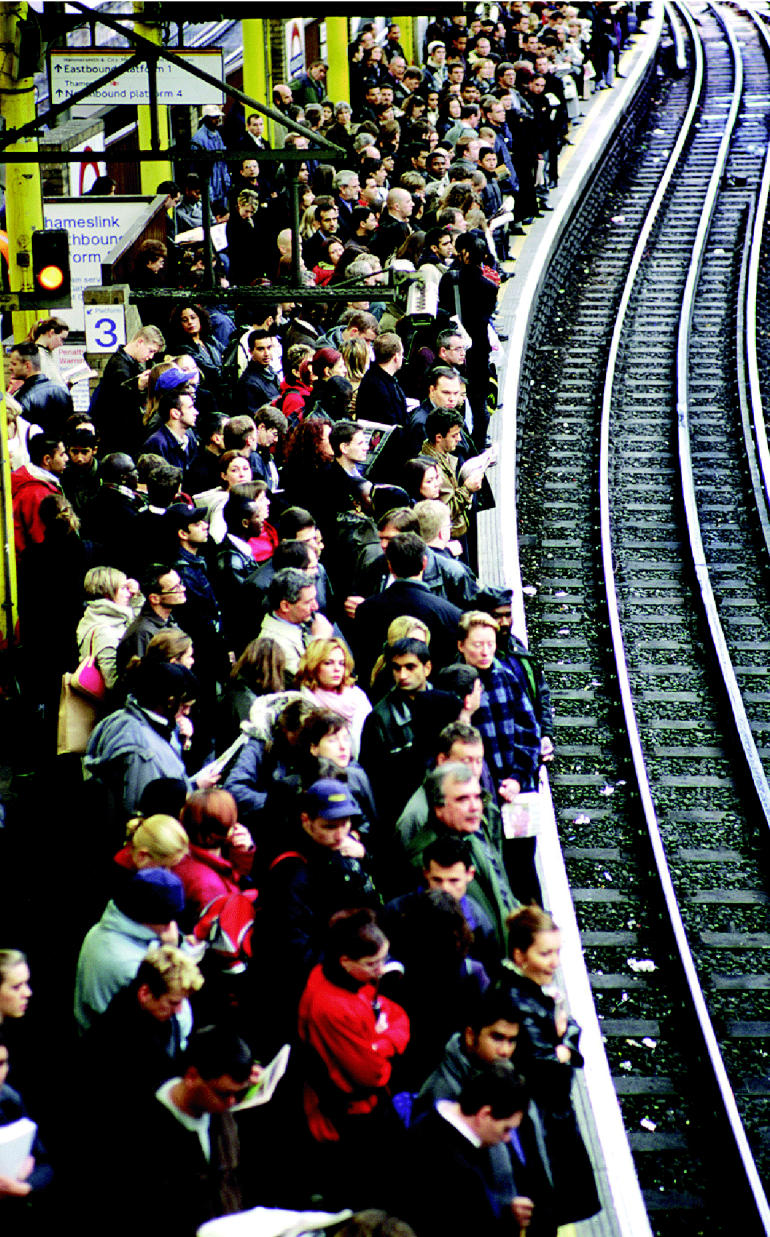


**Figure:**
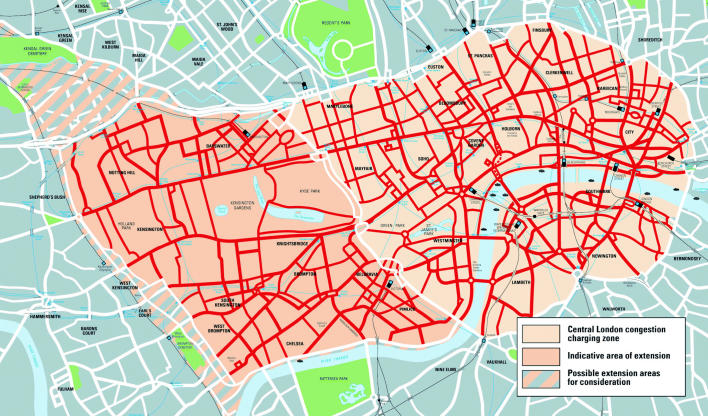
Costs of commuting The city of London utilizes a soon-to-be expanded “congestion pricing” scheme (above), charging drivers for traversing certain areas of the city center. The result is more use of public transit such as the tube train (left, during the morning rush hour).

